# Tunable bound states in the continuum in active metasurfaces of graphene disk dimers

**DOI:** 10.1515/nanoph-2023-0463

**Published:** 2023-12-04

**Authors:** Jose Luis Pura, Juan R. Deop-Ruano, Diego R. Abujetas, Vincenzo Giannini, Alejandro Manjavacas, José A. Sánchez-Gil

**Affiliations:** Instituto de Estructura de la Materia (IEM-CSIC), Consejo Superior de Investigaciones Científicas, Serrano 121, 28006 Madrid, Spain; GdS-Optronlab, Física de la Materia Condensada, Universidad de Valladolid, Paseo de Belén 19, 47011 Valladolid, Spain; Instituto de Óptica (IO-CSIC), Consejo Superior de Investigaciones Científicas, Serrano 121, 28006 Madrid, Spain; Physics Department, Fribourg University, Chemin de Musée 3, 1700 Fribourg, Switzerland; Technology Innovation Institute, Abu Dhabi, United Arab Emirates; ENSEMBLE3 sp. z o.o., Warsaw 01-919, Poland

**Keywords:** graphene, metasurfaces, bound states in the continuum, plasmonics, active metasurfaces

## Abstract

Bound states in the continuum (BICs) in metasurfaces have lately attracted a great deal of attention stemming from their inherent (formally) divergent *Q* factors, which lead to an enhancement of light–matter interaction in two-dimensional geometries. However, the development of plausible means to actively manipulate them remains a major challenge. The use of graphene layers has recently been suggested, employed either as a substrate or a coating that modifies the dielectric environment of the metasurface. Here, instead, we propose to exploit graphene disk dimers supporting in-plane plasmons directly as active meta-atoms in a square array. We prove analytically that both the emergence of a BIC and its *Q* factor can be tuned in an active manner by applying a different external potential to each of the disks in the dimer, thus being formally equivalent to engineering the disk diameters in a passive, geometrically-dependent manner. Moreover, we propose an approach to mitigate the effect of the inherent losses of graphene plasmons based on exploiting the collective behavior of the array, which is achieved by adjusting the lattice parameter so that the wavelength of the BIC mode lies closer to the Rayleigh anomaly.

## 1 Introduction

Metasurfaces (MSs) are artificial devices composed of subwavelength-sized elements that are arranged in a two-dimensional structure. These elements can be designed to strongly interact with light in ways unattainable with naturally occurring materials, resulting in unique and customizable optical properties. MSs present a wide range of potential applications, including imaging [1], sensing [2, 3], telecommunications [4], and energy [5], among many others [6].

Typically, the properties of conventional MSs are fixed during the fabrication process. However, there is a growing interest in developing active MSs that offer the flexibility to modify their characteristics after manufacturing and during device operation, thus enabling the on-demand alteration of their optical response. A wide range of approaches have been explored to actively manipulate the properties of MSs [7–11]. One such approach involves modulating the carrier concentration to modify the dielectric response of the constituent elements. This modulation can be achieved through different means, such as photoexcitation [12] or electrical gating [13]. Another approach relies on the utilization of phase change materials like Ge_2_Sb_2_Te_5_ (GST) [14] and VO_2_ [15, 16]. These materials exhibit the ability to modify their optical properties in response to changes in temperature. By controlling the temperature, the optical properties of MSs incorporating these materials can be adjusted accordingly. Other strategies consider the application of external magnetic fields to achieve magneto-optical control of the response of the system [17–19], or even mechanical actuation by using microelectromechanical systems (MEMS) [20, 21]. By exploring these diverse avenues, researchers aim to enhance the adaptability and controllability of MSs, paving the way for their application in advanced optical devices and systems.

One of the most interesting properties of MSs is their ability to support bound states in the continuum (BICs) under the appropriate conditions [22, 23]. BICs are perfectly confined states that are unable to couple to the radiative continuum, i.e. they cannot radiate or be excited by far-field radiation. As a result, in the absence of nonradiative losses, they formally display a vanishing linewidth and, hence, a diverging *Q* factor. BICs have been observed and studied in the optical-infrared range [24–26], as well as in the THz regime [27, 28]. Indeed, there is great interest in the use of MSs capable of supporting BICs in the THz regime for future applications in information processing and communications [29, 30]. In this context, graphene constitutes a very promising alternative to conventional materials due to its ability to support strong plasmons in this part of the spectrum, which can be tuned by modifying the carrier concentration via, for instance, electrostatic gating [31–34]. These exceptional properties make graphene an ideal platform for manufacturing tunable MSs that support BICs in the THz regime [35–37].

Despite its potential, the use of graphene in this context has mostly been limited to its addition as a substrate or a coating to standard dielectric [38–40], or metallic (typically Au) [41, 42] structures supporting BICs. On the other hand, the optical properties of arrays of graphene disks have been under intensive study in recent years [43–49], but without exploring the possible appearance of BICs in this kind of arrangement.

Here, we study the BICs supported by periodic arrays of graphene microdisks containing two elements per unit cell and characterize their optical properties. We show that the electrostatic tuning of the plasmons supported by the graphene disks allows us to control the frequency and the *Q* factor of the BIC resonance using an external electric field. Furthermore, we propose an approach to mitigate the limitations imposed by the nonradiative losses of graphene on the *Q* factor of the BIC resonances, which is based on increasing the collective nature of the mode by tuning the lattice parameter of the array. Our results open new avenues for the design and engineering of tunable THz devices, where the frequency and spectral characteristics can be finely adjusted to meet specific application requirements.

## 2 Methodology

**Figure 1:**
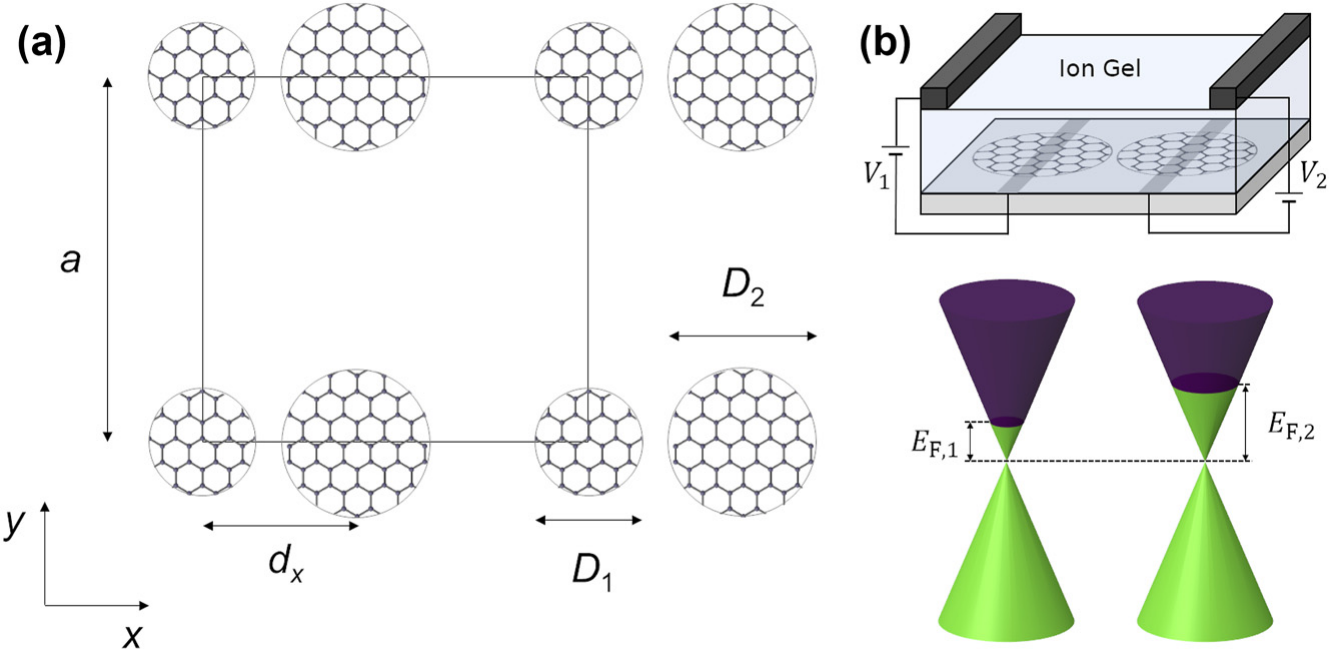
Description of the system under study. (a) Schematics of the square array of graphene microdisk dimers under study, which indicates the lattice parameter *a*, the separation between the disks *d*_*x*_, and their diameters *D*_1_ and *D*_2_. (b) Illustration of a potential implementation of the system using an electrostatic gating approach. The different external potentials applied to the disks result in different Fermi levels, as illustrated in the band diagrams.

The analyzed system consists of a square array of pairs of graphene microdisks with lattice parameter *a*, as depicted in Figure 1a. The disks are separated by a distance *d*_*x*_ = 15 μm < *a*/2 along the *x*-axis. The center-to-center distance *d*_*x*_ is chosen such that the dipolar approximation remains within acceptable limits. The lattice parameter of the array, unless otherwise stated, is set to *a* = 40 μm. We model the disks as point electric dipoles, whose response is encoded in the electric polarizability tensor $\overset{⃡}{\alpha }$ . Taking into account the rotational symmetry of the disks along the *z*-axis, the in-plane diagonal terms of the electrical polarizabilities satisfy $\alpha_{xx}^{\left(i\right)}=\alpha_{yy}^{\left(i\right)}=\alpha^{\left(i\right)}$ , where the superscript *i* takes the values 1 and 2, labeling the two different graphene disks. The remaining elements of the tensor are equal to zero. Following previous works [49], we calculate *α* ^(*i*)^ using the plasmon wave function formalism [33, 50, 51], including the appropriate electrodynamic corrections derived in Ref [49]. Within this approach, as shown in the Supplementary Information S1, the electric polarizability admits an analytical expression that depends on the frequency *ω*, the disk diameter *D*_*i*_, and the Fermi level of the disk *E*_F,*i*_.

In order to analyze the different modes appearing in the array, we employ a coupled dipole model capable of describing the optical response of an infinite array with two disks per unit cell [27, 52–56]. Within this approach, denoting the field induced in each disk of the unit cell as $\psi_{j}^{\left(i\right)}$ , the eigenmodes of the system are obtained from the solution of the following equation [27]

(1) $$\left(1-k^{2}{\overset{⃡}{G}}_{b,jj}\overset{⃡}{\alpha }\right)\left(\begin{array}{c} \psi_{j}^{\left(1\right)}\\ \psi_{j}^{\left(2\right)} \end{array}\right)=0,$$

where

$${\overset{⃡}{G}}_{b,jj}=\left(\begin{array}{cc} G_{b,jj} & G_{jj}^{\left(1-2\right)}\\ G_{jj}^{\left(2-1\right)} & G_{b,jj} \end{array}\right),\;\overset{⃡}{\alpha }=\left(\begin{array}{cc} \alpha^{\left(1\right)} & 0\\ 0 & \alpha^{\left(2\right)} \end{array}\right).$$

In these expressions, *k* = *ω*/*c* is the wave number of light, *G*_*b*,*jj*_ represents the self-interaction of each dipole in the unit cell, and $G_{jj}^{\left(1-2\right)}$ and $G_{jj}^{\left(2-1\right)}$ describe the interaction of dipole 1 with dipole 2, and vice versa, which are equal at normal incidence. Equation (1) can be solved for TE and TM polarization by selecting the index *j* as *y* or *x*. In the following, we analyze in detail the TE case (i.e. *j* = *y*), although a similar derivation can be obtained for the TM mode by replacing ${\overset{⃡}{G}}_{b,yy}$ with ${\overset{⃡}{G}}_{b,xx}$ . Furthermore, to find an analytical expression for the condition under which a BIC occurs, we neglect the nonradiative losses of the graphene disks (see Supplementary Information S2). However, later in the manuscript, we recover them to perform a realistic characterization of the system under study. The solution of Eq. (1) for the TE mode at normal incidence produces the following eigenmodes and eigenvectors

$$\Lambda^{\pm }=k^{2}\left(\frac{1}{\alpha }-G_{b,yy}\right)\pm k^{2}\sqrt{{\left(G_{yy}^{\left(1-2\right)}\right)}^{2}+\frac{{\left(\Delta \alpha \right)}^{2}}{4}},$$

(2) $$\psi^{\pm }=\left(\begin{array}{c} \frac{\Delta \alpha }{2G_{yy}^{\left(1-2\right)}}\mp \sqrt{1+{\left(\frac{\Delta \alpha }{2G_{yy}^{\left(1-2\right)}}\right)}^{2}}\\ 1 \end{array}\right)\approx \left(\begin{array}{c} \mp 1\\ 1 \end{array}\right),$$

where we have introduced $\alpha =2k^{2}{\left(1/\alpha^{\left(1\right)}+1/\alpha^{\left(2\right)}\right)}^{-1}$ representing the average polarization of the disks, and Δ*α* = (1/*α* ^(1)^ − 1/*α* ^(2)^)/*k* ^2^ quantifying their difference. Note that the approximation in the expression for the amplitude of the modes, Eq. (2), becomes exact when the two disks are identical, i.e. Δ*α* = 0. This allows us to label the mode associated with Λ^−^ as the symmetric/in-phase mode and the one corresponding to Λ^+^ as the antisymmetric/out-of-phase mode.

In order to characterize the emergence of BICs in the system, we need to examine the width of the modes. This can be done by analyzing the imaginary part of the corresponding eigenvalue at the resonance frequency. In principle, a symmetry-protected BIC can only occur when the optical responses of the disks are identical, i.e. when the system presents a *C*_2_ symmetry [27, 57]. Furthermore, in the absence of nonradiative losses, it can be demonstrated that [27, 53]

$$Im\left[\frac{1}{\alpha }-G_{b,yy}+G_{yy}^{\left(1-2\right)}\right]=0.$$

Using this relation into the Eq. (2) together with the condition Δ*α* = 0, we have that

$$Im\left[\Lambda^{+}\right]=0,$$

and

$$Im\left[\Lambda^{-}\right]=Im\left[2k^{2}\left(\frac{1}{\alpha }-G_{b,yy}\right)\right]\neq 0.$$

This confirms that, while the width of the symmetric/in-phase mode is always finite, the one corresponding to the antisymmetric/out-of-phase mode vanishes when Δ*α* = 0, becoming a BIC at normal incidence (in absence of nonradiative losses). If Δ*α* ≠ 0, the imaginary part of both eigenvalues never vanishes resulting in two modes with finite lifetime.

To characterize the optical response of the array arising from these modes, in the following, we analyze the extinction produced by the array. As shown in the Supplementary Information S3, this quantity can be calculated as [49]

$$E=\frac{4\pi k}{a^{2}}Im\left(A\right),$$

in terms of the effective polarizability of the array $A={\left({\overset{⃡}{\alpha }}^{-1}-k^{2}{\overset{⃡}{G}}_{b,jj}\right)}^{-1}$ .

## 3 Results and discussion

### 3.1 Array extinction

**Figure 2:**
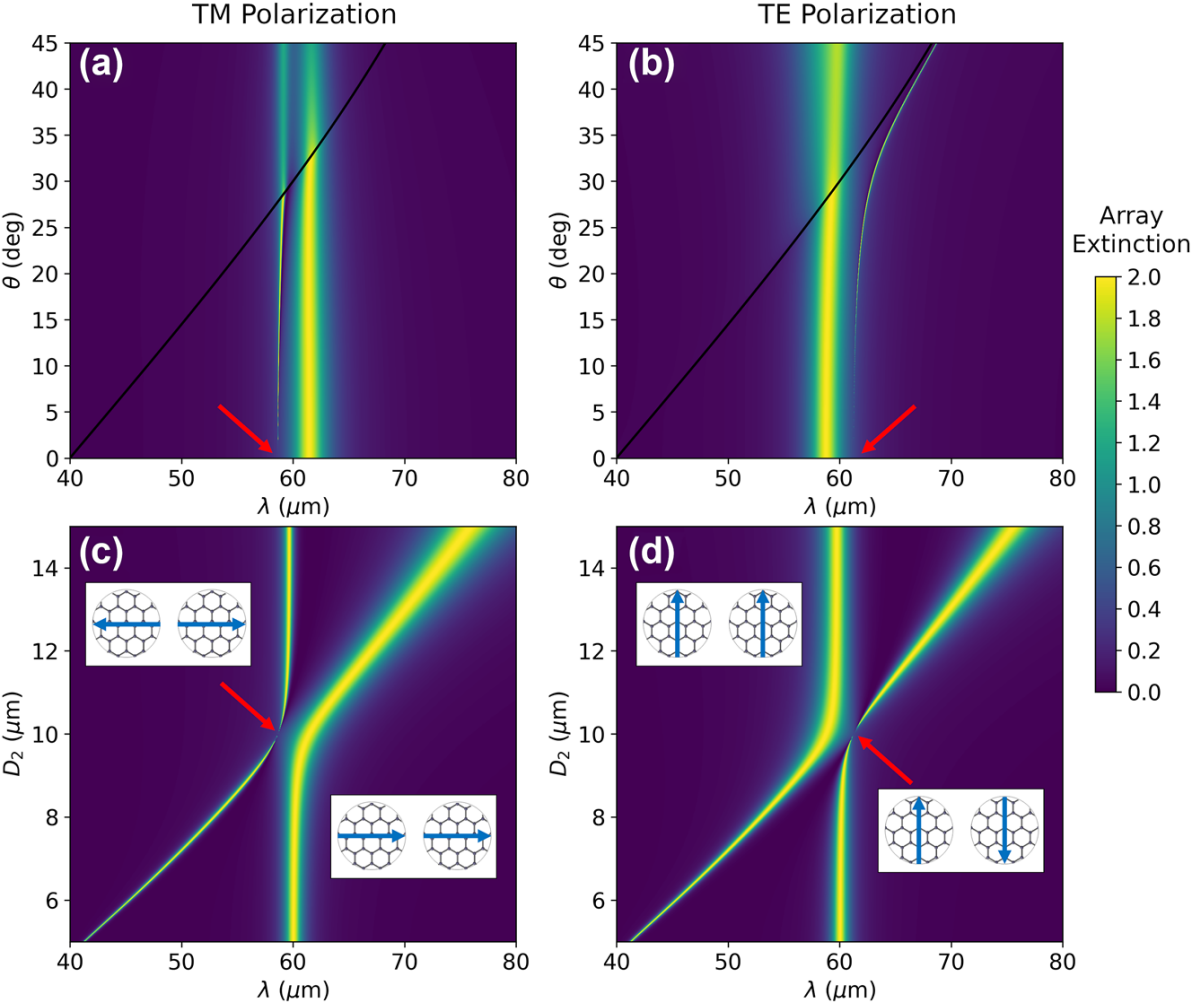
Array extinction. (a and b) Angular dispersion of the array extinction for TM and TE polarizations. We consider an array with two identical graphene disks with diameters *D*_1_ = *D*_2_ = 10 μm. The black line depicts the first Rayleigh anomaly of the system. (c and d) Array extinction for TM and TE polarizations as a function of *D*_2_, with *D*_1_ = 10 μm, calculated at normal incidence. The insets depict the two different modes of the system for TM and TE polarization, while the red arrows signal the location of the BIC. Notice that the BIC appears at normal incidence when both disks are identical. In all of the panels, the Fermi levels of the disks are *E*_F,1_ = *E*_F,2_ = 0.8 eV. Importantly, the nonradiative losses of the graphene disks are neglected.

The extinction of the array with *a* = 40 μm obtained with the coupled dipole model is analyzed in Figure 2. Specifically, Figure 2a and b show the angular dispersion of the TM and TE modes, respectively, for two identical graphene disks with *D*_1_ = *D*_2_ = 10 μm, and *E*_F,1_ = *E*_F,2_ = 0.8 eV. For both polarizations, we observe the two modes associated with the eigenvalues Λ^+^ and Λ^−^. The symmetric modes, which we denote as TM^−^ and TE^−^, display an almost constant spectral width. In contrast, the width of the antisymmetric modes, TM^+^ and TE^+^, is significantly smaller and tends to zero at normal incidence, as expected from the emergence of the BIC (see red arrows). An interesting observation is that, as opposed to the rest of the modes, the TE^+^ mode never crosses the first Rayleigh anomaly of the array, which is indicated by the black line.

Figure 2c and d show the array extinction at normal incidence as a function of *D*_2_, for *D*_1_ = 10 μm and *E*_F,1_ = *E*_F,2_ = 0.8 eV. As expected, the BIC appears only when both graphene disks are identical, i.e. when the system displays *C*_2_ symmetry. When this symmetry is broken, the TM^+^ and TE^+^ modes become quasi-BICs with a finite but significantly large *Q* factor that can be continuously tuned [27, 57]. Importantly, depending on whether *D*_2_ is larger or smaller than *D*_1_, the spectral position of the modes can be tuned or left unchanged by a variation of *D*_2_. In particular, for the TE^+^ mode a smaller value of *D*_2_ results in a negligible shift of the resonance wavelength, while values of *D*_2_ larger than *D*_1_ produce an almost linear variation of the resonance wavelength. The opposite behavior is observed for the TM^+^ mode.

**Figure 3:**
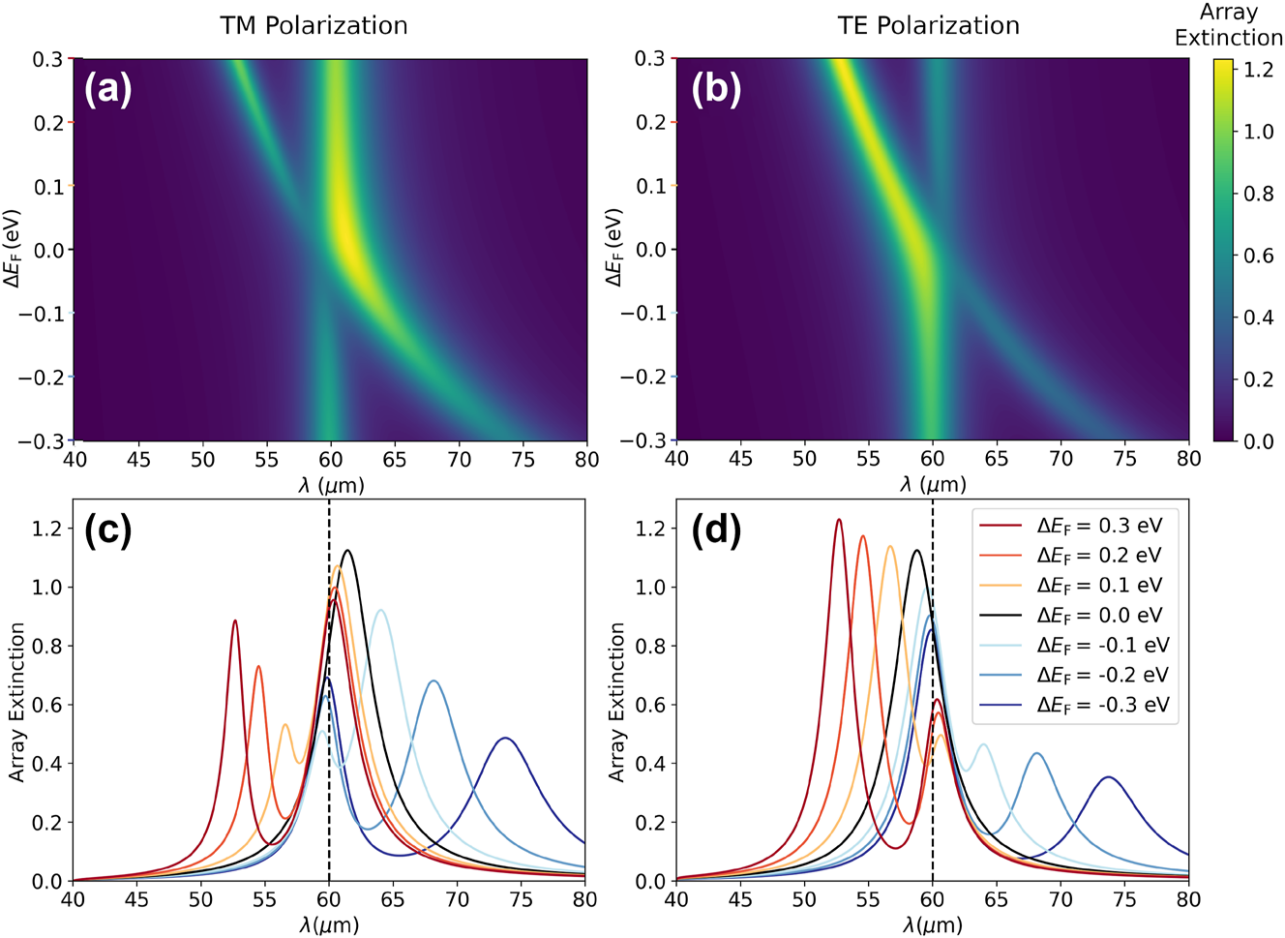
Tunability of the array extinction. (a and b) Array extinction for TM and TE polarizations as a function of Δ*E*_F_ = *E*_F,1_ − *E*_F,2_. We assume *a* = 40 μm, *D*_1_ = *D*_2_ = 10 μm, and *E*_F,2_ = 0.8 eV and include the nonradiative losses of the graphene disks. (c and d) Line cuts of the array extinction at different values of Δ*E*_F_ as indicated in the legend. The black curve corresponds to the case with symmetry condition for the quasi-BIC, i.e. Δ*E*_F_ = 0. The dashed vertical line denotes the resonant wavelength of an array with a single disk per unit cell and separates the positions of the symmetric and antisymmetric modes.

The results of Figure 2, which are obtained neglecting the nonradiative losses of the disks, demonstrate that the system under study can support BICs for the right geometrical configuration. However, one of the most exceptional properties of graphene disks is the possibility of modifying their optical response by tuning the carrier density, which could be achieved using an electrostatic gating approach, as illustrated in Figure 1b. This makes it possible to reach a BIC by tuning the Fermi level of one of the disks, rather than its diameter, thus allowing for active control of the optical response of the system. To explore this possibility, we plot, in Figure 3a and b, the array extinction for TE and TM polarization, respectively. We consider *a* = 40 μm, *D*_1_ = *D*_2_ = 10 μm, *E*_F,2_ = 0.8 eV and vary the value of *E*_F,1_. The results, which are calculated including the nonradiative losses of the graphene disks, are plotted as a function of Δ*E*_F_ = *E*_F,1_ − *E*_F,2_. Importantly, the presence of nonradiative losses prevents the occurrence of a BIC when Δ*E*_F_ = 0. Instead, the resulting quasi-BIC displays a finite width and hence a finite *Q* factor. Nonetheless, for the symmetric configuration Δ*E*_F_ = 0, the quasi-BIC mode still becomes completely dark. This can be observed in Figure 3c and d, where we plot cuts of the array extinction for the different values of Δ*E*_F_ indicated in the legend. When Δ*E*_F_ ≠ 0, the spectrum displays two peaks, corresponding to the symmetric and the antisymmetric modes. These peaks appear one on each side of the dashed line, which indicates the position of the resonance of the array with a single disk per unit cell. However, for Δ*E*_F_ = 0 (black line), the spectrum only displays the resonance corresponding to the symmetric (bright) mode.

### 3.2 *Q* factor

One of the most important magnitudes of an optical resonance is its *Q* factor, which is defined as *Q* = *λ*_peak_/Γ, i.e. the ratio between the spectral position of the mode, *λ*_peak_, and its FWHM, denoted as Γ. We can obtain an estimation of the value of *Q* following an approach similar to that developed in Refs. [49, 53] (see Supplementary Information S4 for further details). The derivation allows us to obtain the following approximation of Γ for the antisymmetric mode

$$\Gamma \approx {\left|\frac{2Im\left(\Lambda^{+}\right)}{\frac{\partial }{\partial \lambda }Re\left(\Lambda^{+}\right)}\right|}_{\lambda =\lambda_{\text{peak}}}.$$

Note that this expression is evaluated at the spectral position of the mode *λ*_peak_.

**Figure 4:**
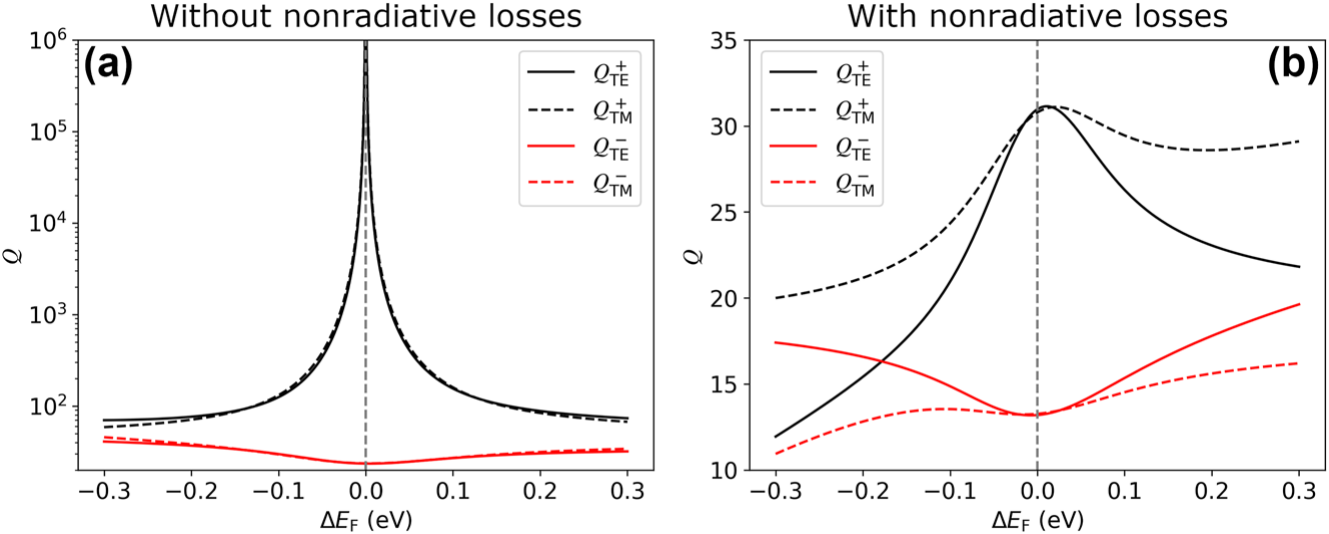
*Q* factor of the symmetric and antisymmetric modes for TE and TM polarizations as a function of the Fermi level detuning. Panels (a) and (b) display the results obtained without and with the nonradiative losses of the graphene disks.

The black curves of Figure 4a display the estimated value of the *Q* factor in the absence of nonradiative losses for the antisymmetric mode with TE, $Q_{\text{TE}}^{+}$ , and TM polarization, $Q_{\text{TM}}^{+}$ . For comparison, the red curves indicate the value of *Q* for the symmetric mode, $Q_{\text{TE}}^{-}$ and $Q_{\text{TM}}^{-}$ . As expected, in the absence of nonradiative losses, the *Q* factor for the antisymmetric mode diverges when we approach the condition Δ*E*_F_ = 0. This confirms the BIC nature of that mode. When the nonradiative losses are included, the values of the *Q* factor are significantly reduced, as shown in Figure 4b, reaching a maximum value of around 30 when Δ*E*_F_ = 0.

**Figure 5:**
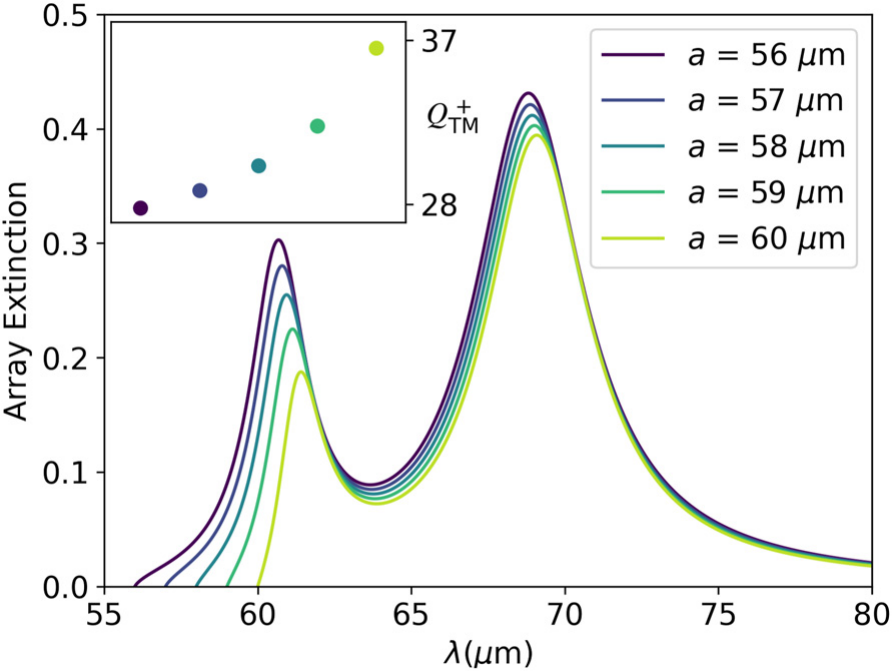
Evolution of the array extinction for TM polarization with the lattice parameter *a*, calculated at normal incidence assuming Δ*E*_F_ = −0.2 eV. The inset shows the value of $Q_{\text{TM}}^{+}$ as a function of *a*.

It is possible to improve the value of *Q* by increasing the lattice parameter of the array *a*. Doing so forces the Rayleigh anomaly to become closer to the resonances of the system, which makes them acquire a more collective behaviour, thus reducing the effect of the nonradiative losses [49, 53, 56, 58, 59]. Figure 5 shows the dependence of the array extinction with the lattice parameter in the range *a* = 56–60 μm, calculated at normal incidence. The results, which are plotted for the TM mode assuming Δ*E*_F_ = −0.2 eV, clearly confirm that the FWHM of both the symmetric and antisymmetric mode are reduced as *a* is increased.

## 4 Conclusions

In summary, we have shown that, when appropriately designed, arrays built from dimers of graphene disks can sustain quasi bound states in the continuum (quasi-BICs). Our results demonstrate that both the spectral position of the quasi-BIC and its *Q* factor can be tuned not only by passively manipulating the geometrical properties of the disks, such as their diameters, but also by actively controlling the carrier density using an externally-applied electric potential. This active approach is especially convenient since, for any given combination of diameters, we can always find the right doping levels such that the array supports a quasi-BIC. Furthermore, we have shown that the moderate *Q* factors arising from the inherent nonradiative losses of graphene plasmons can be improved by engineering the lattice parameter to approximate the Rayleigh anomaly to the spectral location of the quasi-BIC.

Our results provide a new approach to obtain actively tunable quasi-BICs in two dimensional systems. Although, for simplicity, we have focused on a bipartite array, our approach can be readily applied to more complex arrangements of graphene disks or to graphene structures with other shapes. Furthermore, our theoretical scheme could be applied to systems based on other active (electric, magnetic, thermal) materials [8, 9], thus paving the way to exploit the potential of BICs to boost a variety of optical phenomena [24], with applications in non-linear optics, sensing, lasing, to cite a few.

## Supplementary Material

Supplementary Material Details
