# Supplementary material for: Tunable bound states in the continuum in active metasurfaces of graphene disk dimers
Source: Nanophotonics. 2023 Dec 4;12(24):4453–60. doi: 10.1515/nanoph-2023-0463 (PMC11502110; doi:10.1515/nanoph-2023-0463)
Supplement: Supplementary file 1 — Supplementary Material Details [file j_nanoph-2023-0463_suppl_001.pdf]

Tunable Bound States in the Continuum in Active Metasurfaces of Graphene Disk Dimers

Supplementary Information

Jose Luis Pura[1,2,*], Juan R. Deop-Ruano[3], Diego R. Abujetas[4], Vincenzo Giannini[1,5,6], Alejandro Manjavacas[3], and José A. Sánchez-Gil[1,*]

[1]Instituto de Estructura de la Materia (IEM-CSIC), Consejo Superior de Investigaciones Científicas, Serrano 121, 28006 Madrid, email: j.sanchez@csic.es
[2]GdS-Optronlab, Física de la Materia Condensada, Universidad de Valladolid, Paseo de Belén 19, 47011 Valladolid, Spain
[3]Instituto de Óptica (IO-CSIC), Consejo Superior de Investigaciones Científicas, Madrid 28006, Spain
[4]Physics Department, Fribourg University, Chemin de Musée 3, 1700 Fribourg, Switzerland
[5]Technology Innovation Institute, Abu Dhabi, United Arab Emirates
[6]ENSEMBLE3 sp. z o.o., Warsaw 01-919, Poland
[*]joseluis.pura@uva.es

S1 Graphene Electric Polarizability

In order to model the electric polarizability of the graphene disks, we use the plasmon wave function formalism [1, 2, 3, 4]. Within this approach, which is based on the electrostatic solution of Maxwell's equations, the electric polarizability of a graphene disk can be written as

$$\alpha_0 = \frac{D^3\xi^2}{-1/\eta - i\omega D/\sigma}. \tag{S1}$$

Here, $\eta = -0.07249$ and $\xi = 0.85020$ are constants [5], whose value is solely determined by the shape of the structure, D is the diameter of the disk, and σ is the electric conductivity of graphene. For the latter, we adopt a Drude model

$$\sigma = \frac{ie^2}{\pi\hbar^2}\frac{E_\mathrm{F}}{\omega + i\gamma}, \tag{S2}$$

where E_{F} is the Fermi energy, $\gamma = ev_F^2/(\mu E_{\mathrm{F}})$ is the damping coefficient, with $v_F \approx c/300$ being the Fermi velocity of electrons in graphene and μ their mobility. Throughout this work we fix the latter to $\mu = 10^4\,\mathrm{cm}^2/(\mathrm{V\,s})$ [6].

Due to the combination of the large dimensions of the disks under consideration and the particular range of Fermi levels explored, it is necessary to modify the expression of the polarizability given in Eq. (S1) with the appropriate electrodynamic corrections. With these corrections, which have been derived in a previous work [4], the polarizability of disk i becomes

$$\frac{1}{\alpha^{(i)}} = \frac{1}{\alpha_0^{(i)}} - 3\frac{k^2}{D_i} - \frac{2}{3}ik^3,$$

with $k = \omega/c$ being the wave number of light.

S2 Nonradiative Losses

In order to find the precise conditions for which the system under study supports a bound state in the continuum (BIC), in Figure 2 of the main paper, we perform an analysis of its optical response neglecting the nonradiative losses of the graphene disks. The simplest way to remove such losses is by taking $\gamma = 0$ in Eqs. (S2) and (S1). Equivalently, we can define the polarizability of disk i in absence of nonradiative losses as

$$\frac{1}{\tilde{\alpha}^{(i)}} = \mathfrak{Re}\left(\frac{1}{\alpha^{(i)}}\right) - \frac{2}{3}ik^3,$$

which effectively removes the imaginary part of $\alpha_0^{(i)}$ and, hence, the nonradiative losses of the disk.

S3 Effective Polarizability of the Array

Following the usual procedure of the coupled dipole model [7, 8, 9, 10, 11, 12], the electric dipole induced in each graphene disk of the unit cell of the array, $p_j^{(1)}$ and $p_j^{(2)}$, is given by the solution of

$$\left(\overleftrightarrow{\alpha}^{-1} - k^2\overleftrightarrow{G}_{b,jj}\right)\begin{pmatrix} p_j^{(1)} \\ p_j^{(2)} \end{pmatrix} = \begin{pmatrix} \psi_0 \\ \psi_0 \end{pmatrix},$$

where the subscript $j = x, y$ denotes the Cartesian axis corresponding to the polarization of the field incident on the array and ψ_0 its amplitude. By solving this equation, we obtain the effective polarizability of the array

$$\mathcal{A} = \frac{p_j^{(1)} + p_j^{(2)}}{\psi_0} = \frac{1/\alpha^{(1)} + 1/\alpha^{(2)} + 2k^2\left(G_{jj}^{(1-2)} - G_{b,jj}\right)}{\left(1/\alpha^{(1)} - k^2G_{b,jj}\right)\left(1/\alpha^{(2)} - k^2G_{b,jj}\right) - \left(k^2G_{jj}^{(1-2)}\right)^2}. \tag{S3}$$

S4 Q Factor

In order to calculate the Q factor of the different modes supported by the array, we follow a procedure similar to that presented in [4]. Explicitly, we start by expressing the effective polarizability of the array $\mathcal{A}$, derived in Eq. (S3), as

$$\mathcal{A} = \frac{C^+}{\Lambda^+} + \frac{C^-}{\Lambda^-}.$$

The coefficients $C^{\pm} = 1 \mp 1/\delta$ are obtained by simple algebraic manipulations, with $\delta = \sqrt{1 + \Delta\alpha^2/4(G^{(1-2)})^2}$. Then, focusing on the contribution of the antisymmetric mode, we get

$$\mathcal{E}^+ \propto \mathfrak{Im}(\mathcal{A}^+) = \mathfrak{Im}\left(\frac{C^+}{\Lambda^+}\right) = \frac{\mathfrak{Im}(C^+)\mathfrak{Re}(\Lambda^+) - \mathfrak{Re}(C^+)\mathfrak{Im}(\Lambda^+)}{\mathfrak{Re}^2(\Lambda^+) + \mathfrak{Im}^2(\Lambda^+)}.$$

Then, we expand the complex functions C^+ and Λ^+ around the resonance wavelength λ_{peak}, which allows us to calculate the half width at half maximum of the mode, which we denote as $\Delta\lambda$, from the condition $\mathcal{E}^+(\lambda_{\text{peak}} + \Delta\lambda) = \mathcal{E}^+(\lambda_{\text{peak}})/2$. By doing so, and taking into account that $\frac{\partial \mathfrak{Im}(C^+)}{\partial\lambda}\Delta\lambda \ll \mathfrak{Im}(C^+)$, $\frac{\partial \mathfrak{Im}(\Lambda^+)}{\partial\lambda}\Delta\lambda \ll \mathfrak{Im}(\Lambda^+)$, $\frac{\partial \mathfrak{Im}(\Lambda^+)}{\partial\lambda} \ll \frac{\partial \mathfrak{Re}(\Lambda^+)}{\partial\lambda}$, and $\mathfrak{Im}(\Lambda^+)\frac{\partial \mathfrak{Re}(C^+)}{\partial\lambda} \ll \mathfrak{Im}(C^+)\frac{\partial \mathfrak{Re}(\Lambda^+)}{\partial\lambda}$ (with all the expressions evaluated at $\lambda = \lambda_{\text{peak}}$), we obtain two solutions for $\Delta\lambda$

$$\Delta\lambda_{\pm} = \left|\frac{-\mathfrak{Im}(C^+) \pm |C^+|}{\frac{\mathfrak{Re}(C^+)}{\mathfrak{Im}(\Lambda^+)}\frac{\partial}{\partial\lambda}\mathfrak{Re}(\Lambda^+)}\right|_{\lambda=\lambda_{\text{peak}}}.$$

We have verified that one of the solutions underestimates the value of the Q factor, while the other one overestimates it. Therefore, we employ the geometric mean of both solutions to approximate the full width half maximum Γ of the resonance

$$\frac{\Gamma}{2} \approx \sqrt{\Delta\lambda_+\Delta\lambda_-} = \left|\frac{\mathfrak{Im}(\Lambda^+)}{\frac{\partial}{\partial\lambda}\mathfrak{Re}(\Lambda^+)}\right|_{\lambda=\lambda_{\text{peak}}}. \tag{S4}$$

In order to ascertain the validity of this expression, we benchmark it against the FWHM obtained from a fitting of the corresponding extinction peak using the following Fano-like function

$$f(\lambda) = \frac{A}{\pi}\frac{(\lambda - \lambda_{\text{peak}}) + q(\Gamma/2)}{(\lambda - \lambda_{\text{peak}})^2 + (\Gamma/2)^2},$$

where q represents the so-called asymmetry parameter. The results of the benchmark are shown in Figure S1. In particular, we compare the resulting Q factor, defined as $Q = \lambda_{\text{peak}}/\Gamma$. Notice that the results of the fitting become unreliable in the region $|\Delta E_{\text{F}}| \lesssim 0.05\,\text{eV}$.

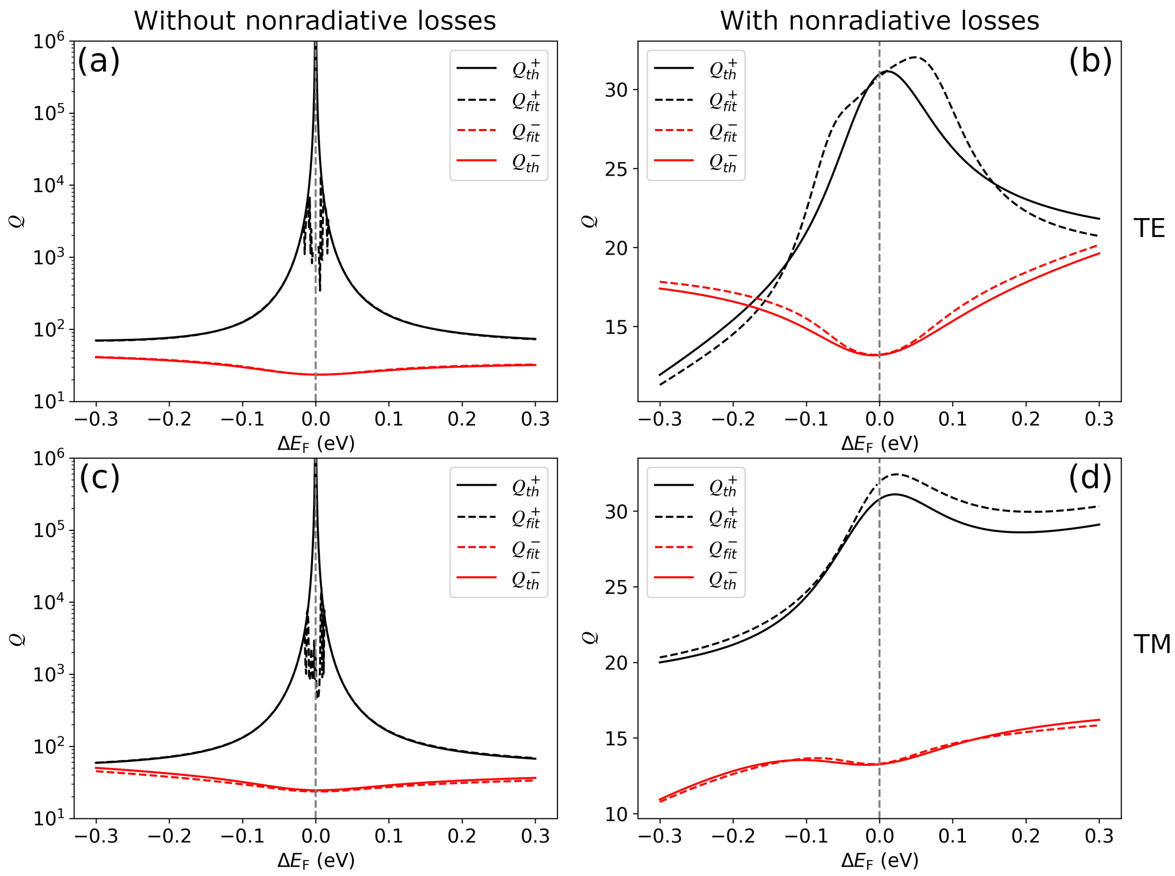

Figure S1: Benchmark of the predictions of Eq. (S4) against the results obtained from the fitting of the corresponding extinction peaks.

References

[1] F. Javier García de Abajo. Graphene plasmonics: Challenges and opportunities. *ACS Photonics*, 1:135–152, 2014.

[2] I. Silveiro, J. M. Plaza Ortega, and F. J. García de Abajo. Plasmon wave function of graphene nanoribbons. *New J. Phys.*, 17:083013, 2015.

[3] Renwen Yu, Joel D. Cox, J. R. M. Saavedra, and F. Javier García de Abajo. Analytical modeling of graphene plasmons. *ACS Photonics*, 4(12):3106–3114, 2017.

[4] Juan R. Deop-Ruano, Stephen Sanders, Alessandro Alabastri, Wilton J.M. Kort-Kamp, Diego A.R. Dalvit, and Alejandro Manjavacas. Optical Response of Periodic Arrays of Graphene Nanodisks. *Phys. Rev. Applied*, 18(4):1, 2022.

[5] Y. Muniz, A. Manjavacas, C. Farina, D. A. R. Dalvit, and W. J. M. Kort-Kamp. Two-photon spontaneous emission in atomically thin plasmonic nanostructures. *Phys. Rev. Lett.*, 125:033601, 2020.

[6] K.I. Bolotin, K.J. Sikes, Z. Jiang, M. Klima, G. Fudenberg, J. Hone, P. Kim, and H.L. Stormer. Ultrahigh electron mobility in suspended graphene. *Solid State Communications*, 146(9):351–355, 2008.

[7] Sebastian Baur, Stephen Sanders, and Alejandro Manjavacas. Hybridization of Lattice Resonances. *ACS Nano*, 12(2):1618–1629, feb 2018.

[8] Diego R. Abujetas, Niels van Hoof, Stan ter Huurne, Jaime Gómez Rivas, and José A. Sánchez-Gil. Spectral and temporal evidence of robust photonic bound states in the continuum on terahertz metasurfaces. *Optica*, 6(8):996, 2019.

[9] Alvaro Cuartero-González, Stephen Sanders, Lauren Zundel, Antonio I. Fernández-Domínguez, and Alejandro Manjavacas. Super- And Subradiant Lattice Resonances in Bipartite Nanoparticle Arrays. *ACS Nano*, 14(9):11876–11887, 2020.

[10] Diego R. Abujetas, Jorge Olmos-Trigo, Juan J. Sáenz, and José A. Sánchez-Gil. Coupled electric and magnetic dipole formulation for planar arrays of particles: Resonances and bound states in the continuum for all-dielectric metasurfaces. *Phys. Rev. B - Condens. Matter Mater. Phys.*, 102(12):125411, sep 2020.

[11] Vladimir R. Tuz and Andrey B. Evlyukhin. Polarization-independent anapole response of a trimer-based dielectric metasurface. *Nanophotonics*, 10(17):4373–4383, 2021.

[12] Lauren Zundel, Alvaro Cuartero-González, Stephen Sanders, Antonio I. Fernández-Domínguez, and Alejandro Manjavacas. Green Tensor Analysis of Lattice Resonances in Periodic Arrays of Nanoparticles. *ACS Photonics*, 9(2):540–550, feb 2022.
